# Temporal variations of bacterial and eukaryotic community in coastal waters—implications for aquaculture

**DOI:** 10.1007/s00253-024-13176-5

**Published:** 2024-06-20

**Authors:** Fulin Sun, Chunzhong Wang, Zhantang Xu, Xingyu Song, Haiping Cui, Zhen Wang, Zhiyuan Ouyang, Xiaoming Fu

**Affiliations:** 1 South China Sea Institute of Oceanology, Chinese Academy of Sciences, Guangzhou, China; 2 State Key Laboratory of Tropical Oceanography, South China Sea Institute of Oceanology, Chinese Academy of Sciences, Guangzhou, China; 3 Sanya Institute of Ocean Eco-Environmental Engineering, Sanya, China; 4 Putian Institute of Aquaculture Science of Fujian Province, Putian, China; 5 Key Laboratory of Tropical Marine Bio-Resources and Ecology, South China Sea Institute of Oceanology, Chinese Academy of Sciences, Guangzhou, China; 6 Nansha Marine Ecological and Environmental Research Station, Chinese Academy of Sciences, Sansha, China

**Keywords:** Aquaculture, Bacterial community, Eukaryotic community, Temporal variations, Potential pathogenic bacteria

## Abstract

**Abstract:**

Despite increased attention to the aquaculture environment, there is still a lack of understanding regarding the significance of water quality. To address this knowledge gap, this study utilized high-throughput sequencing of 16S rRNA and 18S rRNA to examine microbial communities (bacteria and eukaryotes) in coastal water over different months through long-term observations. The goal was to explore interaction patterns in the microbial community and identify potential pathogenic bacteria and red tide organisms. The results revealed significant differences in composition, diversity, and richness of bacterial and eukaryotic operational taxonomic units (OTUs) across various months. Principal coordinate analysis (PCoA) demonstrated distinct temporal variations in bacterial and eukaryotic communities, with significant differences (*P* = 0.001) among four groups: F (January-April), M (May), S (June–September), and T (October-December). Moreover, a strong association was observed between microbial communities and months, with most OTUs showing a distinct temporal preference. The Kruskal–Wallis test (*P* < 0.05) indicated significant differences in dominant bacterial and eukaryotic taxa among months, with each group exhibiting unique dominant taxa, including potential pathogenic bacteria and red tide organisms. These findings emphasize the importance of monitoring changes in potentially harmful microorganisms in aquaculture. Network analysis highlighted positive correlations between bacteria and eukaryotes, with bacteria playing a key role in network interactions. The key bacterial genera associated with other microorganisms varied significantly (*P* < 0.05) across different groups. In summary, this study deepens the understanding of aquaculture water quality and offers valuable insights for maintaining healthy aquaculture practices.

**Key points:**

*• Bacterial and eukaryotic communities displayed distinct temporal variations.*

*• Different months exhibited unique potential pathogenic bacteria and red tide organisms.*

*• Bacteria are key taxonomic taxa involved in microbial network interactions.*

## Introduction

Large-scale aquaculture has emerged as the predominant approach for offshore fish production, while pond and reclamation culture remain the dominant modes of aquaculture, relying on the importation of seawater from offshore sources. The quality of the water, encompassing its biological composition and physicochemical properties, plays a critical role in ensuring the health and sustainability of aquaculture operations. In recent years, frequent environmental calamities have significantly impacted the aquaculture industry, underscoring the importance of maintaining high-quality water in aquaculture.

Microorganisms play an important role in aquaculture, as they play a pivotal role not only in material circulation and energy flow but also in maintaining the stability of the culture environment (Moriarty 1997; Zorriehzahra et al. 2016). Bacteria, in particular, exert a distinctive influence on the intestinal development, nutrition, immune response, and disease resistance of host animals (Balcázar et al. 2006). However, pathogenic bacteria may also reside in the intestinal tract of animals, and their proliferation may disturb the balance of intestinal microbiota, thus leading to compromised host immunity or damage to the intestinal mucosal barrier (Round and Mazmanian 2009). In severe cases, such an imbalance can cause animal diseases and fatalities, which cause substantial losses for aquaculture. Meanwhile, red tides caused by planktonic eukaryotes in the water supply may also impose severe impact on aquaculture (Sun et al. 2020b). Presently, red tide constitutes one of the most common marine calamities and poses a grave threat to aquaculture activities. Certain red tide species may secrete toxins or decompose into toxic substances, thereby altering the community structure of the marine ecosystem and harming the ecological environment of mariculture and fisheries (Fleming et al. 2011; Matsuyama and Shumway 2009). Hence, it is of great importance to investigate the microbial composition of the aquaculture environment, particularly for preventing the outbreak of pathogenic bacteria and red tide organisms.

To date, the significance of water quality remains insufficiently understood, with the composition of microorganisms and pathogens in water sources often overlooked during practical production. Addressing this issue, the present study aims to investigate a seawater reclamation area by conducting long-term observations of microbial communities in nearshore water (i.e., aquaculture water source). The study aims to analyze temporal variations in bacterial and eukaryotic community compositions, the characteristics of potentially pathogenic bacteria and red tide organisms, and the interaction patterns and key taxa among microbes in different months. The study's outcomes will serve as a scientific reference for aquaculture health.

## Materials and methods

### Study area and sample collection

Water samples were collected from an aquaculture zone in Putian city, China, during different months: May to December 2019, January to December 2021, and January to May 2022. All culture ponds in this area shared the same seawater source, entering the system through an inlet (Sun et al. 2019). The samples were taken in the nearshore water area close to the culture zone, at a depth of 0.5 m, using a plexiglass water sampler. Specifically, 1000 ml of water samples underwent filtration with polycarbonate membranes featuring a pore size of 0.2 μm (EMD Millipore, USA). The filtration membranes were then placed in 1.5 ml sterile centrifuge tubes and stored in liquid nitrogen. Samples DNA extraction was performed using the PowerWater DNA Isolation Kit (MoBio, United States). The extraction process adhered to the DNA extraction protocol, conducted under sterile conditions to prevent contamination.

### Illumina MiSeq Sequencing of bacteria and eukaryote

DNA from the above samples underwent amplification using specific primers: 515F (5’–GTG CCA GCM GCC GCGGTA A–3’) and 806R (5’–GGA CTA CHV GGG TWTCTA AT–3’) (Caporaso et al. 2011) for the 16S rRNA gene. For eukaryotes, the amplification of 18S rRNA was conducted with the specific V9 primers 1380F (5’-CCCTGCCHTTTGTACACAC-3’) and 1510R (5’-CCTTCYGCAGGTTCACCTAC-3’) (Amaral-Zettler et al. 2009). The polymerase chain reaction (PCR) used 15 µL of Phusion® High-Fidelity PCR Master Mix (New England Biolabs). PCR conditions were as follows: initial denaturation at 95 °C for 3 min, followed by 30 cycles of denaturation at 95 °C for 30 s, annealing at 55 °C (for bacteria) and 57 °C (for eukaryotes) for 30 s, extension at 72 °C for 45 s, and a final extension at 72 °C for 10 min. After purifying the PCR products, library preparation was carried out using the TruSeq® DNA PCR-Free Sample Preparation Kit (Illumina, USA). These libraries were then loaded onto an Illumina NovaSeq 6000 Sequencer (Illumina, USA) to generate the sequences.

### Sequences data analysis

Paired-end reads obtained from Illumina sequencing underwent removal of barcodes and primers before assembly using FLASH (version 1.2.11). The resulting reads were filtered to remove low-quality reads and chimeric sequences using Chimera Check. High-quality reads were then clustered into OTUs with a sequence similarity of 97% using VSEARCH (version 1.9.6). Representative sequences of the OTUs were classified using the Ribosomal Database Program (RDP) classifier (version 2.2) with a confidence threshold of 0.8, based on the SILVA Database (version 132). The representative sequence was assigned by performing a BLAST search against the Silva_123 16S rRNA database and the V9_PR2 18S rRNA database. Based on taxonomic information derived from species annotations, potential pathogenic bacteria and red tide species associated with aquaculture were identified.

In this study, the R project (version 3.3.1) was used to perform PCoA with unweighted unifrac distances. The resulting data were analyzed to identify significant differences in bacterial and eukaryotic communities between groups, using the Wilcoxon rank-sum test with a significance level of *P* < 0.05. Network visualizations depicting bacterial and eukaryotic interactions were generated using Cytoscape software (version 3.7.0). Bacterial genera with Spearman coefficient absolute values ≥ 0.7 and correction *P* values ≤ 0.05 were selected. Each node represents a genus, and its color represents a phylum. Edges color indicate significant correlations (negative and positive) between nodes.

## Results

### 16S rRNA sequencing data analysis

**Fig. 1:**
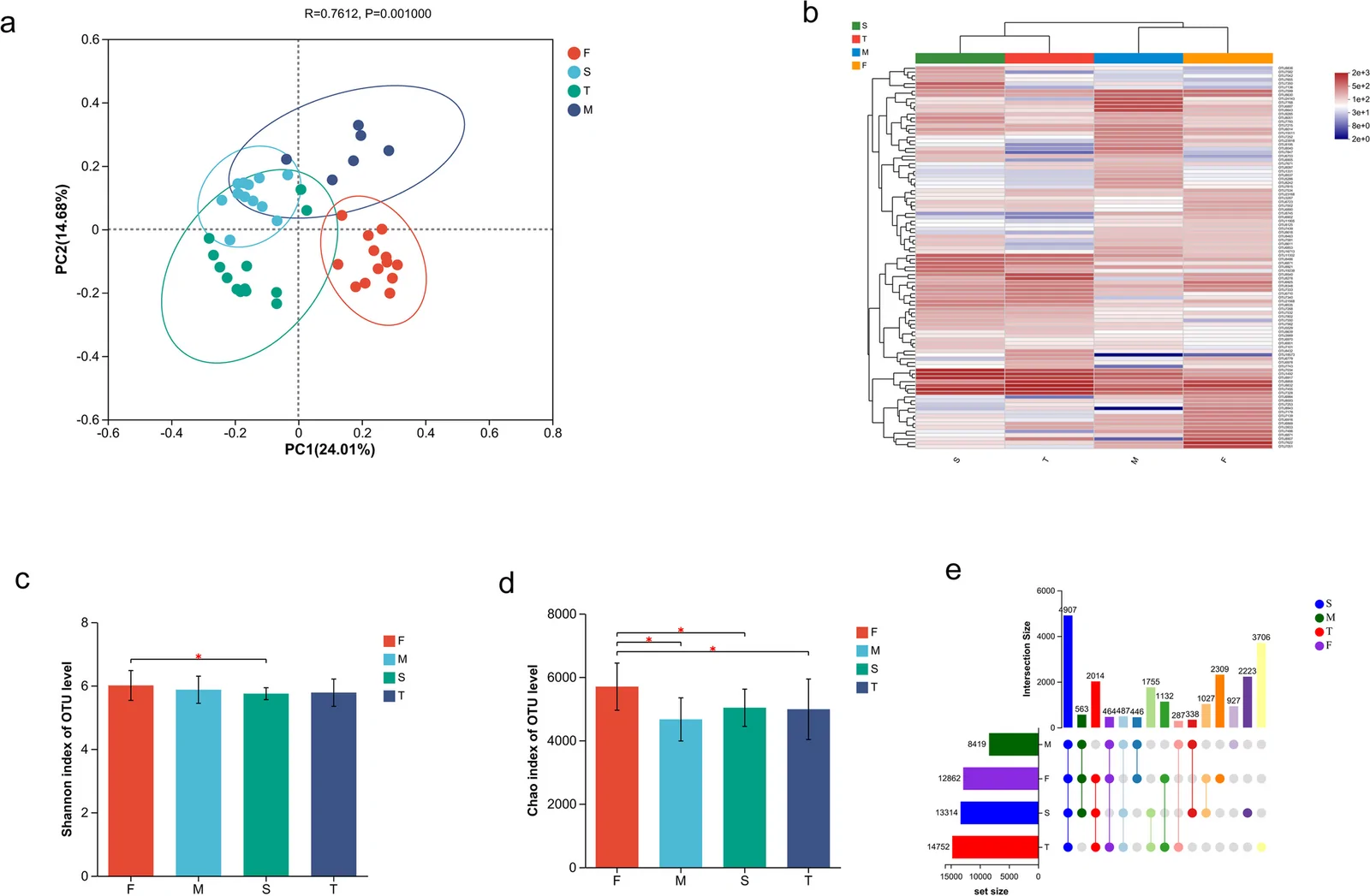
Analysis of the bacterial community structures in the water source. **a** PCoA analysis of the bacterial community at OTU level; **b** Cluster analysis of dominant OTUs among groups; **c** Shannon index; **d** Chao index; **e** UpSet Venn diagram analysis of OTU numbers in groups. F (January-April), M (May), S (June–September), and T (October-December)

In this study, a total of 4,400,314 sequences and 23,658 OTUs were extracted from all samples. PCoA and cluster analysis, depicted in Fig. 1a and b, revealed significant differences (ANOSIM, *P* = 0.001) in bacterial communities among the samples. Based on substantial variations in β diversity, the samples were classified into four distinct groups denoted as F (January-April), M (May), S (June–September), and T (October-December). Furthermore, the F (January-April) and M (May) groups exhibited marked distinctions from the S (June–September) and T (October-December) groups.

Diversity and richness assessments (Fig. 1c and d) revealed notable differences in bacterial diversity of F, M, and S groups, as indicated by the Shannon index, with group F exhibiting the highest value and group S the lowest. Moreover, the Chao index indicated significant richness differences among F, M, S, and T groups, with the highest richness observed in group F and the lowest in group M. The UpSet Venn diagram analysis in Fig. 1e revealed that 4,907 OTUs (21.7%) were common to all four groups. In contrast, 2,309 OTUs (10.2%) were exclusively observed in group F, 927 OTUs (4.1%) were specific to group M, 2,223 OTUs (9.8%) were unique to group S, and 3,706 OTUs (16.4%) were identified solely in group T.

### Bacterial community composition and potential pathogenic bacteria

**Fig. 2:**
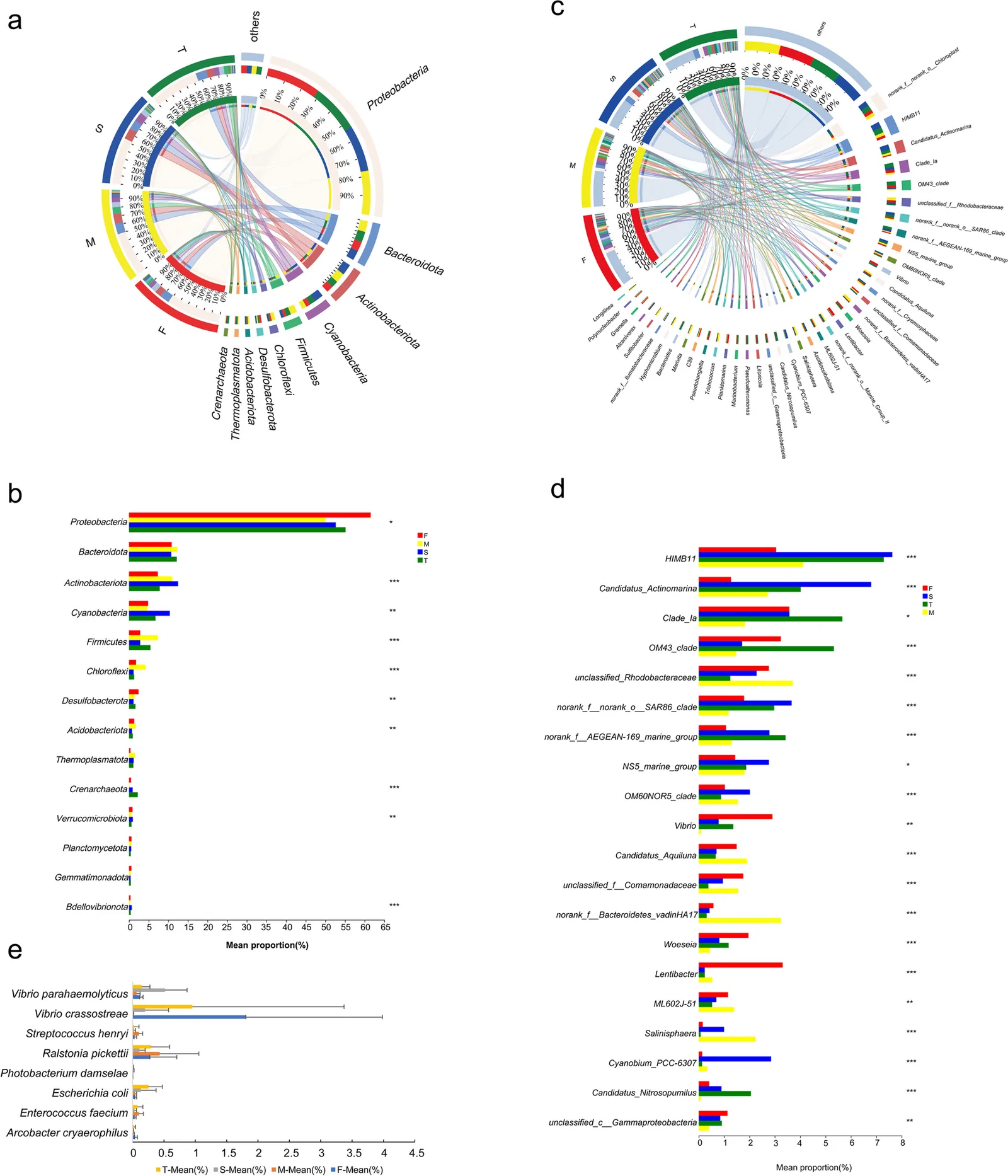
Bacterial communities in the water source. Dominant bacterial taxa at the phylum (**a**) and genus (**c**) levels; Statistical analysis of differences in bacteria at the phylum (**b**) and genus (**d**) levels (Kruskal–Wallis H test, *p* < 0.05); (**e**) temporal variations (*p* < 0.05) in the abundance of potentially pathogenic bacteria. F (January-April), M (May), S (June–September), and T (October-December)

The investigation involved a comparison of bacterial taxa abundance among different groups, based on taxonomic information. Analysis of the bacterial taxa present in the water revealed a higher abundance of *Proteobacteria*, *Bacteroidota*, *Actinobacteriota*, *Cyanobacteria*, and *Firmicutes* (Fig. 2a). Kruskal–Wallis H test (*P* < 0.05, Fig. 2b) revealed that the F group had a significantly higher abundance of *Proteobacteria* (61.5%) and *Desulfobacterota* (2.4%) compared to the other groups. On the other hand, the M group had significantly higher levels of *Firmicutes* (7.4%), *Chloroflexi* (4.2%), and *Acidobacteriota* (1.7%) compared to other groups. The S group, however, was found to have a higher abundance of *Actinobacteriota* (12.5%) and *Cyanobacteria* (10.4%).

The results demonstrated that there were noteworthy dissimilarities (*P* < 0.05) in the dominant genera between the samples (Fig. 2c and d). Specifically, the dominant genera observed in the water samples were HIMB11, *Candidatus Actinomarina*, *SAR11 clade Ia*, *OM43 clade*, *NS5 marine group*, *Lentibacter*, and *Vibrio*. Wallis H test (p < 0.05) determined that *Lentibacter* (3.3%), *Vibrio* (2.90%), and *Planktomarina* (2.4%) exhibited considerably greater prevalence in the F group relative to the other groups. In contrast, the M group exhibited significantly higher abundance of *Trichococcus* (2.2%), *Marivita* (2.0%), and *Salinisphaera* (2.2%) than the other groups. The S group was characterized by a high abundance of *HIMB11* (7.6%), *NS5 marine group* (2.8%), *Candidatus Actinomarina* (6.8%), and *Cyanobium* (2.9%). Furthermore, bacterial genera such as *SAR11 clade Ia* (5.7%), *OM43 clade* (5.3%), *Pseudoalteromonas* (2.2%), *Candidatus Nitrosopumilus* (2.1%), and *Bacteroides* (2.0%) exhibited significantly higher abundance in the T group when compared to the other groups.

Figure 2e illustrated the significant temporal variations (*P* < 0.05) in the abundance of potentially pathogenic bacteria. The abundance of *Vibrio crassostreae* was significantly higher in the F group compared to the other groups. In the M group, high abundance of *Ralstonia pickettii* was observed. Moreover, the abundance of *Vibrio parahaemolyticus* was significantly higher in the S group compared to the other groups. In addition, the T group was characterized by a high abundance of *Escherichia coli* and *Vibrio crassostreae*.

### 18S rRNA sequencing data analysis

**Fig. 3:**
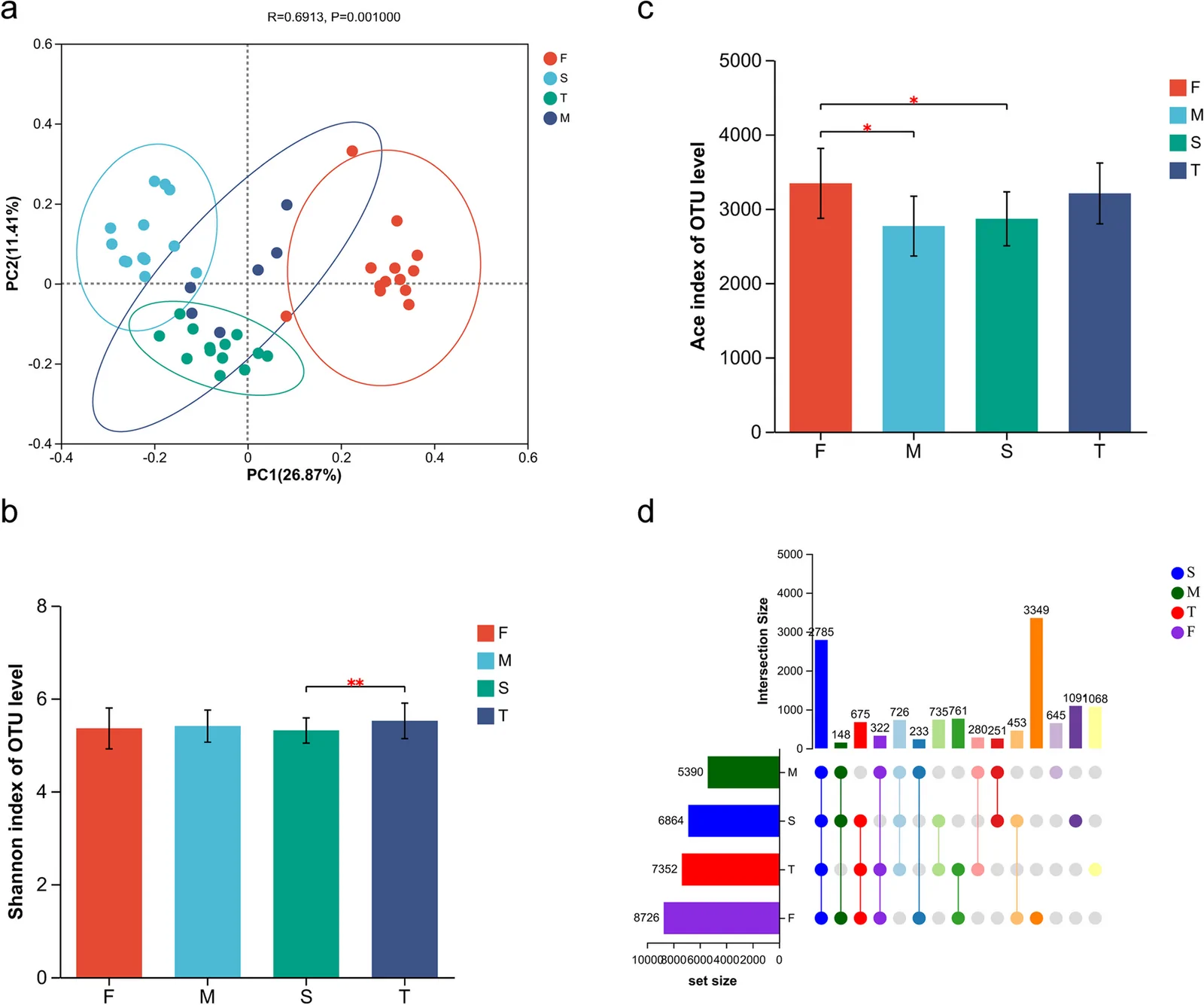
Analysis of the eukaryotic community structures in the water source. **a** PCoA analysis of the eukaryotic community at OTU level; **b** Shannon index; **c** Ace index; **d** UpSet Venn diagram analysis of OTU numbers in samples. F (January-April), M (May), S (June–September), and T (October-December)

In this study, a total of 4,488,534 sequences and 14,562 OTUs were extracted from all the samples. PCoA (Fig. 3a) revealed significant differences (ANOSIM, *P* = 0.001) in the eukaryotic communities among the samples at the OTU level. These samples were also categorized into four groups based on significant differences in β diversity, denoted as F (January-April), M (May), S (June–September), and T (October-December). Additionally, similar to the bacterial community, the eukaryotic community of groups F (January-April) and M (May) were significantly different from those of groups S (June–September) and T (October-December). Diversity analysis revealed that there were significant differences (*P* < 0.01) of eukaryotic OTUs in the Shannon index between groups S and T, with group T showing a higher Shannon index than group S (Fig. 3b). Additionally, Ace index demonstrated significant differences (*P* < 0.05) in eukaryotic OTUs among groups F, M, and S, with group F displaying the highest richness and group M showing the lowest richness (Fig. 3c). Further analysis using the UpSet Veen diagram (Fig. 3d) indicated that 2,785 (20.6%) of the OTUs were common among the four groups, while 3,349 (24.8%), 645 (4.8%), 1,091 (8.1%), and 1,068 (7.9%) of OTUs were unique to groups F, M, S, and T, respectively.

### Eukaryotic community composition and potential red tide organisms

**Fig. 4:**
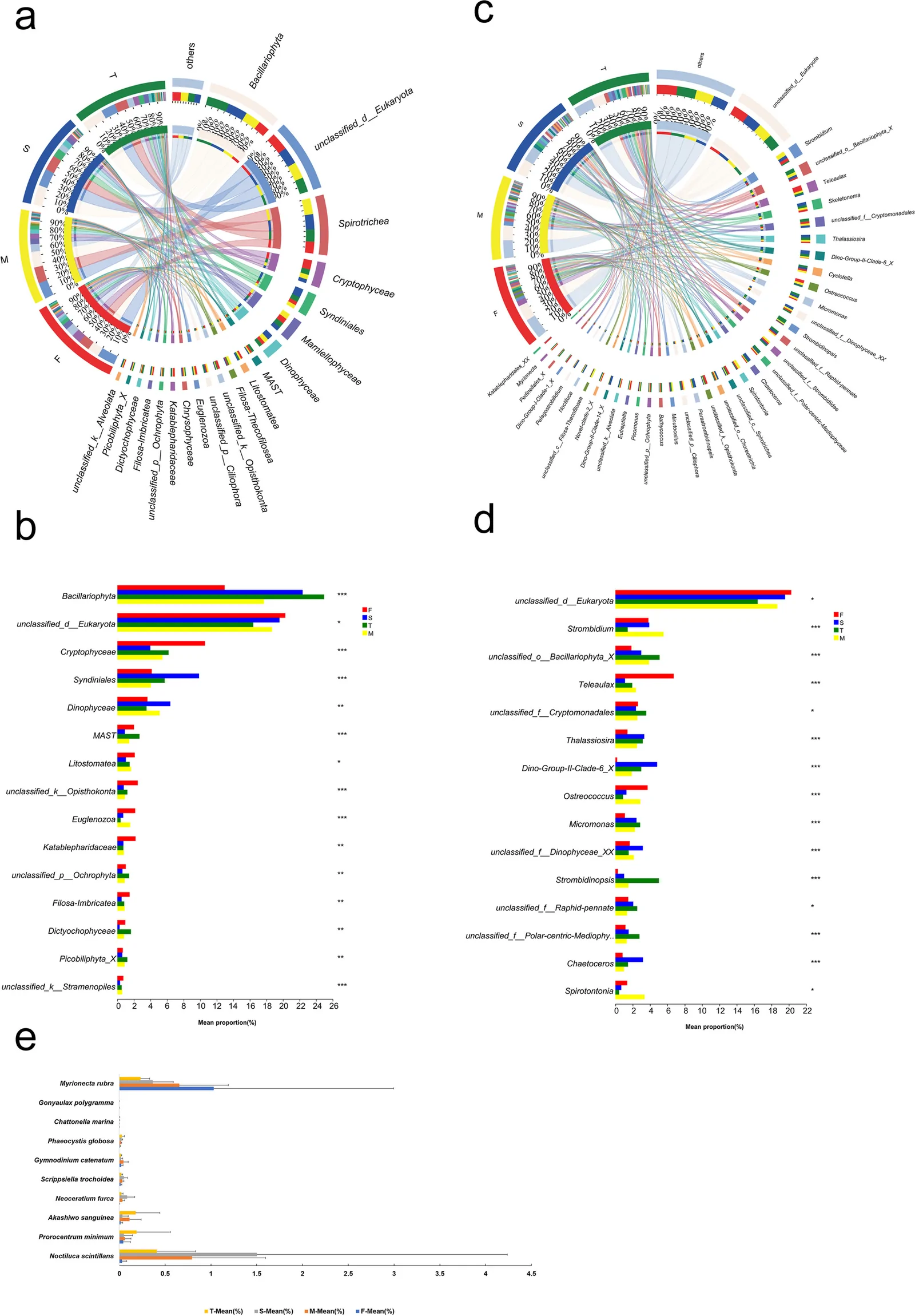
Eukaryotic communities in the water source. Dominant eukaryotic taxa at the phylum (**a**) and genus (**c**) levels; Statistical analysis of differences in eukaryote at the phylum (**b**) and genus (**d**) levels (Kruskal–Wallis H test, *p* < 0.05); (**e**) Temporal variations (*p* < 0.05) in the abundance of red tide organisms. F (January-April), M (May), S (June–September), and T (October-December)

According to taxonomic information, the eukaryote present in the aquatic environment exhibited a greater abundance of *Bacillariophyta*, *Spirotrichea*, *Cryptophyceae*, *Mamiellophyceae*, *Syndiniales*, *Dinophyceae*, and *MAST* (Fig. 4a). The Kruskal–Wallis H test revealed that Cryptophyceae (10.6%), Katablepharidaceae (2.2%), Euglenozoa (2.2%), and Litostomatea (2.1%) had higher abundance in F group, which showed a statistically significant difference (*P* < 0.05, Fig. 4b) with other groups. Furthermore, *Spirotrichea* (18.2%), *Mamiellophyceae* (6.4%), and *Filosa-Thecofilosea* (2.7%) exhibited a significantly higher abundance in group M compared to the other groups. Group S was characterized by a high abundance of *Syndiniales* (9.9%) and *Dinophyceae* (6.4%), whereas group T was distinguished by a higher abundance of *Bacillariophyta* (25.0%) and *MAST* (6.3%).

The results displayed notable dissimilarities (*P* < 0.05) of dominant genera among the samples (Fig. 4c and d). The principal genera identified in the water samples were *Strombidium*, *Teleaulax*, *Skeletonema*, *Thalassiosira*, *Dino-Group-II-Clade-6*, *Cyclotella*, *Ostreococcus*, and *Micromonas*. The Wallis H test (*P* < 0.05) revealed that *Teleaulax* (6.7%), *Ostreococcus* (3.7%), *Eutreptiella* (1.8%), and *Katablepharidales* (1.8%) exhibited considerably higher abundance in group F in comparison to other groups. *Strombidium* (5.6%), *Spirotontonia* (3.4%), and *Minutocellus* (1.7%) were notably more abundant in group M than other groups. Additionally, group S was characterized by a high abundance of *Dino-Group-II-Clade-6_X* (4.8%), *Thalassiosira* (3.3%), *Chaetoceros* (3.2%), *Parastrombidinopsis* (2.9%), *Dino-Group-II-Clade-14_X* (2.7%), and *Pelagostrobilidium* (1.7%). Eukaryotic genera, including *Strombidinopsis* (5.0%), *Micromonas* (2.85%), *Cyclotella* (2.6%), *Bathycoccus* (1.5%), and *Picomonas* (1.2%), exhibited considerably high abundance in group T.

The results presented in Fig. 4e indicate significant temporal variations (*P* < 0.05) in the abundance of certain red tide species. *Myrionecta rubra* exhibited significantly higher abundance in group F compared to other groups, while *Noctiluca scintillans* and *Myrionecta rubra* were distinguished by its high abundance in group M. Furthermore, the abundance of *Noctiluca scintillans*, *Neoceratium furca*, and *Scrippsiella trochoidea* were significantly greater in group S than in the other three groups. In contrast, group T was characterized by a high abundance of *Prorocentrum minimum* and *Akashiwo sanguinea*.

### Network analysis of bacteria and eukaryote

**Fig. 5:**
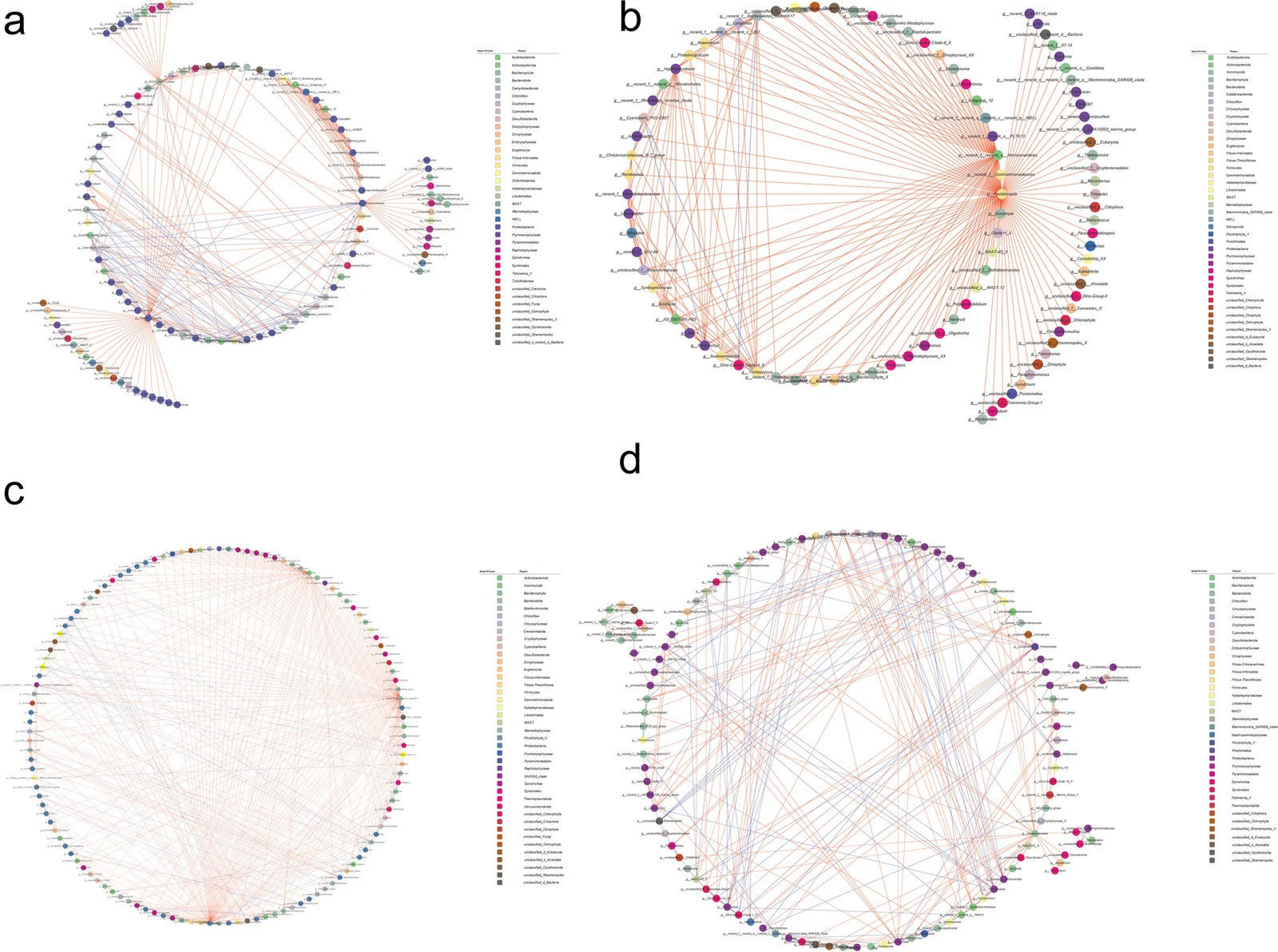
OTU network analysis of the relationships among the bacteria and eukaryotes in group F (January-April)(**a**), M (May)(**b**), S (June–September)(**c**), and T (October-December)(**d**). Each node represents a genus; node colors represent different taxa; the blue lines represent negative correlations; the red lines represent positive correlations

The network diagram in Fig. 5 illustrated intricate interactions between eukaryotic and bacterial communities. Through statistical analysis (*P* < 0.05), the network diagram revealed notable findings in four distinct groups. In group F, there were 56 negative correlations and 190 positive correlations; group M displayed 11 negative correlations and 228 positive correlations; group S showed 73 negative correlations and 335 positive correlations, while group T displayed 109 negative correlations and 182 positive correlations. These observations provided insight into the complex relationships between eukaryotes and bacterial communities.

The key genera that were closely associated with other microorganisms exhibit significant temporal variations and dependent on the group under consideration. Bacteria were the predominant microorganisms in these associations. In group F (Fig. 5a), the top genera most frequently linked with other microorganisms included *Sva0996 marine group*, unclassified *Rhodocyclaceae*, *Glaciecola*, *norank Desulfobulbaceae*, *Cyanobium*, *NS3a marine group*, *no rank Ilumatobacteraceae*, and *Yoonia-Loktanella*. In group M (Fig. 5b), the key genera were most closely associated with other microorganisms comprised *Paracoccus*, *Fastidiosipila*, *Limnobacter*, *no rank Aminicenantales*, *Trichococcus*, *Hyphomicrobium*, *Syntrophomonas*, and *Smithella*. In group S (Fig. 5c), the top genera that were most frequently linked with other microorganisms included *Lactobacillus*, *Tropicimonas*, *hgcI clade*, *Ostreococcus*, *Hyphomicrobium*, *Dino-Group-I-Clade-4_X*, *Dino-Group-II-Clade-14_X*, and *Bacteroides*. Finally, in group T (Fig. 5d), the key genera most commonly associated with other microorganisms were *Synechococcus*, *Candidatus Puniceispirillum*, *OM43* clade, *Porticoccus*, *Litoricola*, *Luminiphilus*, *Tropicimonas*, *SAR86 clade*, and *MAST*.

## Discussion

### Temporal patterns of bacterial and eukaryotic communities

The study's findings indicate that both bacteria and eukaryotes display similar temporal variation patterns, which manifested through changes in α diversity, OTU abundance, and β diversity. Notably, the shifts in microbial communities were not continuous, as evidenced by the separation of communities along the first PCoA axis between the periods of January-May and June-December (Figs. 1a and 3a). Previous investigations have consistently reported temporal shifts in marine microbial communities, attributing these changes to environmental factors (Bunse and Pinhassi 2017; Fuhrman et al. 2015; Gilbert et al. 2012)**.**

According to the network analysis presented in Fig. 5, bacteria play a crucial role in the succession of microbial communities regardless of the month. This implies that, among the major interacting groups, bacteria demonstrate the highest levels of activity and responsiveness compared to eukaryotes. However, the specific bacterial genera exhibiting strong associations with each other undergo significant changes in response to months, indicating their sensitivity to environmental factors. Importantly, alterations in the taxa and abundance of key microorganisms can potentially trigger community-wide changes through complex interactions. These observations are consistent with previous studies (Hunt and Ward 2015; Needham and Fuhrman 2016).

### Temporal variation of dominant bacteria and potential pathogens and their impact on aquaculture

The study reveals notable temporal variations in the structure of bacterial communities. Each group exhibit unique compositions of dominant bacterial genera and potential pathogens, reflecting the impact of environmental changes.

The dominant bacterial populations identified from January to April include *Vibrio*, *Lentibacter*, and *Planktomarina*. *Vibrio crassostreae* stands out as a potential dominant pathogenic bacterium. *Vibrio*, a prevalent group of marine bacteria, showcases metabolic flexibility and plays important roles in utilizing various carbon, nitrogen, and phosphorus substrates (Eiler et al. 2007). *Lentibacter*, observed to exhibit a high level of protein expression in the absorption and metabolism of nitrogenous dissolved organic matter (DOM) metabolites, dominate during the winter season (Han et al. 2021). *Planktomarina* has the ability to generate supplemental metabolic energy through anaerobic photosynthesis and CO dehydrogenase utilization (Giebel et al. 2019). The months of January to April are relatively cold, and the survival strategies of these bacteria are tailored to their development and nutrient utilization under oligotrophic conditions. As the most prevalent potential pathogenic bacterium, *Vibrio* has often been detected in the aquaculture environment and in the intestines of healthy and diseased animals (Sun et al. 2020a, 2022; Sun and Xu 2021). Pathogenic *Vibrio* can lead to significant mortality of cultural animals, causing major losses in revenue. *Vibrio crassostreae*, part of the *Splendidus* clade, stands out as one of the primary bacterial pathogens affecting marine bivalves (Le Roux et al. 2016; Travers et al. 2015). It has the potential to cause successive mass mortality in juvenile oysters (Petton et al. 2015). This finding holds crucial implications for disease prevention in the investigated area, which is a significant oyster production region.

May stand out as a distinctive month, marking the transition between seasons and exhibiting a bacterial composition significantly different from other months. The prominent bacterial genera included *unclassified_f__Rhodobacteraceae*, *Salinisphaera*, *Trichococcus*, and *Marivita*, with *Ralstonia pickettii*, a potential pathogen, showing the highest abundance. These bacteria are closely related to algae in marine and aquaculture environment. The *Rhodobacteraceae* family is known for its diverse metabolic activities, encompassing aerobic anoxygenic photosynthesis, sulfur oxidation, carbon monoxide oxidation, and DMSP demethylation (Brinkhoff et al. 2008). This family likely played a crucial role in maintaining the health of the culture system (Lin et al. 2017). *Marivita*, frequently observed in high abundance in aquaculture environments (Sun et al. 2020b, 2021, 2019), may contribute to nitrogen transformation and removal. *Ralstonia pickettii* has been identified as a potential pathogen affecting hybrid striped bass (Fowler et al. 2021). Moreover, it is a significant human pathogen associated with infections such as osteomyelitis and meningitis, potentially posing a threat to seafood safety (Ryan and Adley 2014).

The predominant bacteria genera observed from June to September were *HIMB11*, *NS5_marine_group*, *Candidatus Actinomarina*, *OM60*, and *Cyanobium*_PCC-6307. Notably, *Vibrio parahaemolyticus* was the pathogenic bacteria with the highest abundance during this period. The composition of these dominant bacteria mirrors the characteristics of intense sunlight and high temperatures typical of summer. The elevated contribution of members such as *HIMB11* and *OM60* in warmer months could be attributed to their utilization of alternative energy supply pathways, such as bacterial chlorophylls and protein rhodopsins (Durham et al. 2014; Spring and Riedel 2013). The discovery of a novel rhodopsin in *Candidatus Actinomarina* suggests a photoheterotrophic lifestyle, which could significantly impact carbon cycling (Ghai et al. 2013). The *NS5 marine group* encodes a variety of degraded CAZymes, indicating their capacity to degrade high molecular weight dissolved organic matter (DOM) (Bennke et al. 2016; Priest et al. 2022). The presence of these bacteria plays a crucial role in carbon cycling. *Cyanobium*_PCC-6307 is abundant in June–September, while other months are very low abundance. *Cyanobium*_PCC-6307 is sensitive to increased temperature and sufficient light, potentially leading to cyanobacterial blooms. *Vibrio parahaemolyticus* is a Gram-negative bacterium found in marine and estuarine environments worldwide. Food poisoning caused by *Vibrio parahaemolyticus* typically occurs in summer (June–October) and is positively correlated with water temperature, primarily related to various types of seafood including crabs, shrimps, shellfishes, lobsters, fish, and oysters (Wang et al. 2015).

During the 10–12-month observation period, the predominant bacterial genera included *HIMB11*, *SAR11 clade Ia*, *OM43 clade*, *Candidatus_Nitrosopumilus*, and *AEGEAN-169_marine_group*. Noteworthy findings identified *Escherichia coli* and *Vibrio crassostreae* as potential pathogens due to their elevated abundance. The *SAR11* clade exhibits optimized nutrient uptake even under extremely low nutrient concentrations (Eiler et al. 2009). The *OM43* clade of *Betaproteobacteria*, crucial in C1 metabolism and carbon cycling (Jimenez-Infante et al. 2016; Morris et al. 2006), is ubiquitously present in marine environments. *Escherichia coli*, recognized as common Gram-negative opportunistic pathogens in both human and animal gut environments (Mariani et al. 2021), serves as an indicator of water pollution. Pathogenic *Escherichia coli* can induce diseases in various aquatic animals, leading to significant economic losses in the aquaculture industry. Furthermore, the abundance of *Vibrio crassostreae* remained higher over the 10–12-month period, consistent with observations from 1 to 4 months. This suggests that cooler periods of the year contribute to an increase in the abundance of this bacteria.

### Characteristics of temporal changes in red tide species

As with bacterial communities, eukaryotic communities also have significant temporal characteristics, especially red tide species exhibit month-specific patterns. Although phytoplankton plays a crucial role in marine ecosystems due to its involvement in the material cycle (Arrigo 2004), certain groups of phytoplankton can generate harmful algal blooms (HABs), which can negatively impact both marine ecosystems and human health (Anderson et al. 2002). Compared to other periods, the species *Myrionecta rubra* exhibited the highest abundance in aquatic environments during the months of January through April. The predominant species, *Mesodinium rubrum*, is responsible for blooms across numerous coastal ecosystems (Sanders 1995). Despite *M. rubrum* being recognized as a nontoxic species, its blooms can pose potential risks to aquaculture industries (Hayes et al. 1989).

The dominant red tide species were *Myrionecta rubra* and *Noctiluca scintillans* in May, and the dominant red tide species were *Noctiluca scintillans* in June to September. *Noctiluca scintillans* influences phytoplankton composition through voracious feeding, particularly on diatoms (Crawford et al. 1997). The excretion of ammonia by *Noctiluca scintillans* during grazing, particularly the release of intracellular high ammonia concentrations following cell death, may lead to fish mortality and toxicity in other organisms. The widespread negative effects of *Noctiluca scintillans* and their bloom have made their ecophysiology a prominent area of research (Baliarsingh et al. 2016). The dominant red tide species in 10–12 months were *Prorocentrum minimum* and *Akashiwo sanguinea*. *Prorocentrum minimum*, a common dinoflagellate known for its blooms in coastal environments, is capable of causing harm to humans through shellfish poisoning. The adverse impacts associated with blooms can range from fish to shellfish mortality in aquaculture and may be attributed to indirect biomass effects (e.g., low dissolved oxygen) as well as toxic effects (Heil et al. 2005). *Akashiwo sanguinea*, a frequent bloom-forming species worldwide (Du et al. 2011; Yang et al. 2012), has been shown to be associated with mortality in laboratory-based abalone larvae (Botes et al. 2003). Changes in estuarine fish communities have also been observed during red tide proliferation of *Akashiwo sanguinea*, with significant declines in fish density, abundance, and biomass (Amorim Reis-Filho et al. 2012).

In conclusion, the present study examined the changes in the microbial community in the coastal water during distinct months. Furthermore, this study analyzed in detail the interaction patterns between microorganisms, and the potential impact of pathogenic bacteria and red tide organisms on aquaculture. This study significantly advances our knowledge of the microbial ecology in coastal water, enriched our understanding of water safety in aquaculture, thereby offering a valuable reference for the promotion of healthy aquaculture.

## Data Availability

The data presented in this study were upload to the NCBI Sequence Read Archive Database under the BioProject number PRJNA985906.
